# Isoflavones and PPAR Signaling: A Critical Target in Cardiovascular, Metastatic, and Metabolic Disease

**DOI:** 10.1155/2010/153252

**Published:** 2011-02-24

**Authors:** Rakesh P. Patel, Stephen Barnes

**Affiliations:** ^1^Department of Pathology, University of Alabama at Birmingham, 1918 University Boulevard, Birmingham, AL 35294, USA; ^2^Botanicals Center for Age-Related Disease, Purdue University-University of Alabama at Birmingham, USA; ^3^Department of Pharmacology and Toxicology, University of Alabama at Birmingham, 1918 University Boulevard, Birmingham, AL 35294, USA; ^4^Center for Nutrient-Gene Interaction, University of Alabama at Birmingham, 1918 University Boulevard, Birmingham, AL 35294, USA

## Abstract

Isoflavone intake through foods and dietary supplements has both health advocates and critics. The latter come from a concern about the estrogenic effects of isoflavones in certain species. However, careful removal of isoflavones and other estrogens from the diet of rodents leads to the metabolic syndrome. These results suggest that isoflavones have other mechanisms of action, potentially those involving regulation of fatty acid metabolism via the nuclear receptors PPAR**α** and PPAR**γ**. The goal of this paper was to examine the evidence for isoflavone/PPAR signaling and to identify diseases in which such signaling would have an important impact. It is therefore of note that investigators using a chemical structure approach to discover PPAR ligands identified isoflavones as the best structures in the library of compounds that they tested. Future studies will involve careful identification of the underlying mechanisms whereby isoflavones have their action via PPAR signaling.

## 1. Introduction

The importance of plant estrogens (*phytoestrogens) *in the human diet has become a topic of great interest [1], as well as dispute [2]. The principal phytoestrogens in the American and Western European diets are the isoflavones in soy foods [3, 4]. It is noteworthy that soy protein is widely used for animal diets both in commercial food production and for animals in research studies. In the latter, it has been realized by several investigators that isoflavones have significant physiological effects. Many toxicologists have been concerned that the estrogenic properties of isoflavones could lead to infertility [5]. Such a connection was first observed in sheep infertility in Western Australia which was attributed to the red clover (*Trifolium pratense*) that they consume. Red clover contains large amounts of isoflavones [6]. Similar infertility effects were observed in captive cheetahs [7], although this may be related to the failure of the cat family to glucuronidate many xenobiotics [8]. On the other hand, removal of soy from the diets of rats in chemoprevention experiments led to an increase in incidence of chemically induced mammary tumors [9]. Similarly, soy improved the blood pressure of spontaneously hypertensive rats on a high-salt diet [10] and ameliorated the cold sensitivity of mice with gene knockouts of the first members of the *β* oxidation of long-chain fatty acids [11]. Many such examples of the disparate effects of isoflavones have been reported which stem, in part, from a lack of understanding of biological mechanisms of action of isoflavones in individual species. 

**Figure 1:**
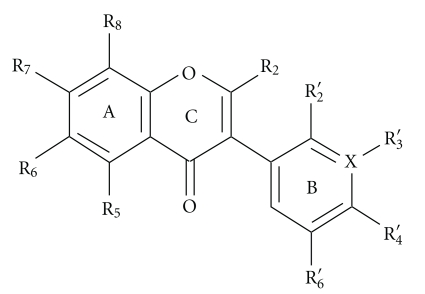
The isoflavones: generic 7-hydroxy-benzopyran-4-one ligands for PPAR*γ*. Isoflavones have a B-ring aryl substituent in the 3-position and a Δ^2-3^double bond. Common isoflavones are daidzein (R_5_ = R_6_ = R_8_ = H, R_7_ = R_4_′ = OH, R_2_′ = R_3_′ = R_5_′ = H′), genistein (R_6_ = R_8_ = H; R_5_ = R_7_ = R_4_′ = OH; R_2_′ = R_3_′ = R_5_′ = H′), formononetin (R_5_ = R_6_ = R_8_ = H; R_7_ = OH; R_2_′ = R_3_′ = R_5_′ = H; R_4_′ = OCH_3_), and biochanin A (R_6_ = R_8_ = H; R_5_ = R_7_ = OH; R_2_′ = R_3_′ = R_5_′ = H; R_4_′ = OCH_3_). The chemical library search also showed that the atom at position-3 in the B-ring can be either carbon or nitrogen.

In this paper, we discuss several lines of emerging evidence implicating isoflavones as activators of PPAR*α* and PPAR*γ*. Indeed, we hypothesize that isoflavone-dependent activation of PPAR*α* and PPAR*γ* signaling is key to understanding how these compounds affect multiple pathophysiological processes. Intriguingly, a study employing structure-based virtual screening with induced fit locking analysis for identifying novel PPAR*γ* ligands revealed that out of a natural product library comprising 200 compounds isoflavones were the optimal PPAR*γ* ligands [12]. We have shown that modification of isoflavones by nitration and/or chlorination, which may occur in vivo, forms novel products with altered efficacy for PPAR*γ* activation [13, 14]. Additionally, a recent comprehensive structure-activity relationship study demonstrated that the 7-hydroxy-benzopyran-4-one structure, which comprises the core isoflavone (and other flavonoids) structure (Figure 1), is key for PPAR activation [15]. Selective modification of this core can form molecules with dual PPAR*α*- and PPAR*γ*- ligand-binding activity [15]. From this perspective, isoflavones and their biological metabolites may provide the template for the next generation of PPAR agonists. Isoflavones are naturally occurring compounds and are safely ingested in amounts up to 100 mg/day. This is an exciting area of investigation and underscores the possibility that these compounds could be used therapeutically with a low occurrence of unwanted side effects. This paper comprises summaries of several aspects of isoflavones: their biochemistry and chemistry, their dietary intake, bioavailability and metabolism, their association with prevention of chronic disease, and their mechanisms of action, with special emphasis on PPAR signaling.

## 2. Biosynthesis and Chemistry of Isoflavones

Isoflavones are members of the huge family of plant polyphenols [16]. The polyphenols include bioflavonoids (e.g., quercetin, catechins, proanthocyanidins) and stilbenoids (resveratrol). Bioflavonoids consist of many classes. Depending on the position of the aromatic B-ring substituent on the heterocyclic C-ring, they can be broadly separated into flavonoids and isoflavonoids. Both are derived from a common precursor, phenylalanine. Following the formation of the flavonoid ring system, the aromatic B-ring migrates from the 2-position to the 3-position catalyzed by an enzyme restricted to tropical leguminous plants. Edible plants containing the highest concentrations of isoflavones are soybeans (*Glycine max* Merrill) [17], kudzu root (*Pueraria lobata*) [18], and the American groundnut (*Apios americana)* [19]. 

The isoflavones in each of these plants are principally glycoside conjugates of daidzein (7,4′-dihydroxyisoflavone) and genistein (5,7,4′-trihydroxyisoflavone). In soy, the conjugates are the 7-*O*-*β*-D-glucopyranosides with additional esterification on the 6′′-position of the glucose moiety [20, 21]. The conjugate groups are removed either by fermentation (to make miso, soy paste, and tempeh) [3] or by intestinal hydrolysis induced by enzymes in the wall of the intestine (lactose phlorizin hydrolase) [22] or by bacteria. In the kudzu root, C-glucoside conjugates of isoflavones (e.g., puerarin, daidzein 8-C-*β*-glucopyranoside) predominate [18]. These are absorbed and excreted without hydrolysis, probably by Na^+^-dependent glucose transporter systems.

## 3. Dietary Intake of Isoflavones

In the Western diet, exposure to isoflavones comes mostly from the use of soy protein to impart useful characteristics to foods such as low-fat dairy and bakery products, soups, doughnuts, hamburger buns, canned fish, and whole turkeys [23]. In addition, vegetarians and those seeking low-fat diets consume soy foods such as soy milk, tofu, and textured vegetable protein. Athletes wanting a high-protein/low-fat diet use isolated soy protein. The average consumer has a daily intake of 1-2 mg isoflavones [24], giving rise to plasma concentrations from 20 to 150 nM. Those consuming 1-2 soy meals a day (20–40 mg isoflavones) have plasma concentrations ranging from 200 to 3000 nM [25]. This wide range of plasma concentrations is typical of many orally ingested therapeutics and represents differences in uptake from and metabolism in the gut, as well as differences in tissue metabolism and urinary and fecal excretion. Isoflavones are also available as over-the-counter dietary supplements nominally containing 50 mg per pill. This enables considerably higher isoflavone intakes. Zeisel and his colleagues have reported phase 1-dose escalation studies where daily doses of >1,000 mg soy isoflavones were used without reported significant hazards [26, 27].

**Figure 2:**
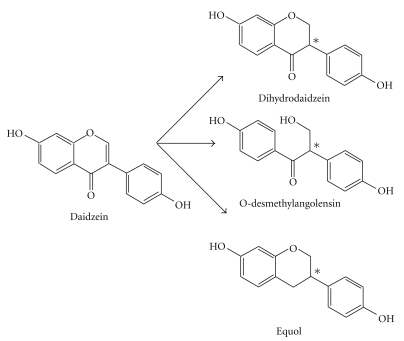
Metabolites of daidzein. Daidzein is converted by anaerobic bacteria in the large intestine to several metabolites, dihydrodaidzein, O-desmethylangolensin, and equol. Each of these metabolites has a chiral center at C-3 due to the reduction of the Δ^2-3^double bond (marked with a star*). Equol is found as its S-(-)-equol enantiomer. The chirality of the other daidzein metabolites is not known.

The isoflavones in the blood, as for physiological steroids and many other xenobiotics, are principally *β*-glucuronides, with lesser amounts of sulfate esters and only low (10–100 nM) concentrations of their aglycone forms [28]. Isoflavones also undergo metabolism in the large intestine (Figure 2), and the bacterial products such as dihydrodaidzein (DHD), O-desmethylangolensin (ODMA), and S-(-)equol enter the circulation [29]. Whereas DHD and ODMA are present in most subjects, only 20–30% of people studied producing S-(-)equol [30, 31]. The discussion above is presented to underscore the importance of appreciating the range of concentrations achieved *in vivo* together with the knowledge that effects of isoflavone consumption may in fact be mediated by their derivatives from intestinal bacterial and/or host cell metabolism, in understanding their mechanisms of action. 

In the next sections, we select some of the diseases that have been shown to be modulated by isoflavones and examine the potential sites of involvement of PPAR signaling and other mechanisms of action.

## 4. Association with Chronic Diseases: Cellular and Animal Models

### 4.1. Isoflavones and Cardiovascular Disease

**Figure 3:**
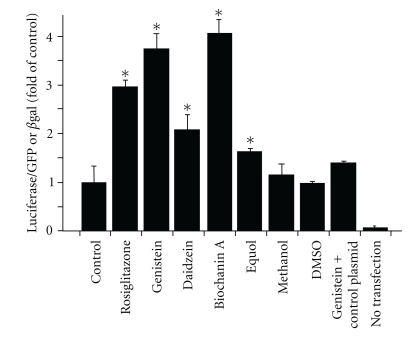
Isoflavones activate the PPAR*γ* promoter: cultured endothelial cells were transfected with plasmids containing the promoter response elements for PPAR*γ* (PPRE) or PPRE-negative plasmids and then exposed to indicated isoflavones (1 *μ*M) and vehicle controls. Data are expressed as fold of the control (i.e., relative to no isoflavone-treated cells) and are means ± SEM (*n* = 3–6). *Different from control, *P* ≤ .005. They illustrate the ability of isoflavones to stimulate transcription of PPAR*γ*-regulated genes. Genistein, daidzein, and biochanin A (4′-methoxygenistein) have promoter activities comparable to rosiglitazone (figure reproduced with permission by the *Journal of Nutrition*).

Consumption of isoflavones is associated with protection against atherosclerosis, a chronic disease of the vessel wall that underlies the development of many acute cardiovascular disease events including myocardial infarction and stroke [32–34]. These observations are supported by experimental studies in diverse animal models of atherosclerosis showing that dietary isoflavones can inhibit the disease [35–37]. Interestingly, if isoflavones are administered only in the latter stages of disease, the protective effects are lost suggesting that these polyphenols target the early events of atherosclerosis [38]. Less clear are the mechanisms by which isoflavones inhibit atherosclerosis. The two general hypotheses are that these compounds are antioxidants and/or modulate specific signaling pathways related to inflammation in the vasculature that affects the disease [39]. With antioxidant effects, the concept has been that by scavenging reactive species, which would otherwise promote oxidative damage, isoflavones prevent atherosclerosis. The most cited example in this case is the inhibition of low-density lipoprotein oxidation, formation of which is central in atherogenesis [40]. More recent evidence suggests the hypothesis that isoflavones modulate vascular disease by affecting signaling pathways. In this paradigm, low (submicromolar) concentrations of isoflavones activate the specific signaling pathways that regulate cellular responses to inflammation. Two candidate pathways defined to date which meet this criterion are activation of ER*β* and that of PPARs [41, 42]. We focus the discussion in this paper on PPARs and note that activation of PPAR*α*, or –*γ*, has been viewed mainly from the perspective of the regulation of genes that control lipid and glucose metabolism [43]. However, emerging data suggest critical roles in modulating vascular inflammatory and immune responses also [44–48]. For example, PPAR*γ* ligands decrease atherosclerotic lesion size in experimental models [49]. The anti-inflammatory effects of PPARs appear to be restricted to the *α* and *γ* isotypes, and from the perspective of controlling endothelial function, PPAR*γ* ligands inhibit cytokine-dependent expression of adhesion molecules (although these responses are dependent upon cell type, nature of the inflammatory stimulus, and specific ligand used) [44, 48]. With respect to isoflavones, cell and animal studies have shown these compounds to be agonists for PPAR*α*- and PPAR*γ*-dependent pathways (see below). For example, the antidiabetic effects of isoflavones are associated with PPAR*γ* activation in macrophages [49], and with respect to vascular inflammation specifically our published studies show that isoflavones activate PPAR*γ* in the endothelium and in turn results in an inhibition of monocyte rolling and adhesion, a key step in inflammation [13, 14] (Figure 3).

### 4.2. Cancer

Little consideration has been given by the cancer research community to possible roles of isoflavone-directed PPAR signaling [50]. Nonetheless, genistein has been shown to lower the production of prostaglandin E2 by MDA-MB-231 human breast cancer cells and to reduce invasiveness of these cells [51]. The effect of eicosapentaenoic and docosahexaenoic acids in activating PPAR*γ* was dependent on genistein [52]. Effects of isoflavones on lipid signaling may be an important aspect of carcinogenesis and tumor invasiveness.

### 4.3. Lymphangioleiomyomatosis

This rare lung disease affects 1 in 100,00 women [53]. It is caused by migration of uterine smooth muscle cells to the lung where they form cysts and cause loss of lung function. Many of the women have mutations in tuberin (TSC1) and harmartin (TSC2) that form the tuberous sclerosis protein complex [54]. The TSC1/TSC2 complex is a critical player in the control of mTOR, a master regulator of cellular metabolism. The migration of ELT-3 cells to the lungs in a rodent model of lymphangioleiomyomatosis is driven by 17*β*-estradiol [55]. There is a concern that the isoflavones may mimic this action of estrogen [56]. However, a recent study on estrogen proliferation of ELT-3s cell also showed that genistein blocked this action of 17*β*-estradiol [57]. Importantly, genistein's inhibitory effect was in turn attenuated by the PPAR*γ* inhibitor GW9662 [57]. This underscores the likelihood that the action of isoflavones in mammals including man is multi-factorial and that PPAR signaling is a target of the isoflavones.

### 4.4. Metabolic Syndrome

There is an extensive literature going back to 2001 linking soy and its isoflavones to lipid metabolism and the metabolic syndrome. Harmon and Harp showed that genistein inhibited the proliferation and differentiation of 3T3-L1 cells, a preadipocyte cell line [58]. Genistein also increased lipolysis in these cells [58]. These investigators also demonstrated that genistein blocked the DNA binding and transcriptional activity of the CCAAT-/enhancer-binding protein beta by promoting the production of C/EBP homologous protein [58]. This in turn impacted PPAR*γ* protein expression [58]. A differential effect of genistein was observed in mesenchymal progenitor cells and revealed opposing effects of estrogen receptor and PPAR*γ* pathways [59]. At low genistein concentrations, the estrogen-like effect was observed, whereas at micromolar concentrations, PPAR*γ* activation predominated [59]. This raises the issue of which of these two effects are observed *in vivo*. Mezei et al. showed that diabetic Zucker rats fed a high isoflavone diet have lower triglyceride and cholesterol concentrations [49]. They also demonstrated that genistein and daidzein significantly increased PPAR*α*- and PPAR*γ*-directed gene expression in murine RAW 264.7 cells [49].

## 5. Isoflavone Mechanisms of Action

Whereas isoflavones and other phytoestrogens were originally studied because of their estrogenic activity in certain species, it has become clear that they have additional mechanisms of action that may override their estrogenic effects. Genistein was identified in 1987 as a potent inhibitor of the epidermal growth factor receptor tyrosine kinase [60]. This was important to the cancer field at that time since genistein, unlike comparable, chemically synthesized tyrosine kinase inhibitors, did not have toxic effects at the doses needed for tyrosine kinase inhibition. Genistein has been widely used as a pharmacological tyrosine kinase inhibitor often without validation that any changes in protein phosphorylation observed on Western blots were due to direct genistein inhibition of phosphorylation as opposed to indirect effects due to a reduction in the parent protein.

Like other polyphenols, many studies have shown that isoflavones can scavenge various reactive oxygen species (RO), reactive nitrogen species (RN) or reactive chlorine species (RCS) that are formed endogenously during the innate immune response, but which also cause tissue injury that leads to the development of acute and chronic inflammatory disease [61–69]. In doing so, the “antioxidant” effect of isoflavones has been proposed to mediate their cytoprotective effects. This concept is supported by human studies showing a decrease in plasma markers of lipid peroxidation after consuming isoflavones [70]. Concerns over the antioxidant hypothesis include the discrepancy between isoflavone concentrations achieved in the circulation (0.1–1 *μ*M) after dietary ingestion and those required to observe a significant inhibition of oxidative damage *ex vivo and in vitro* with the latter typically being ≥10-fold higher. Another consideration is that although the primary reactive species may be scavenged, the products of the reaction and their reactivities must also be considered. With respect to isoflavones, we have shown that upon reacting with lipid peroxyl radicals (which inhibits lipid peroxidation), the corresponding isoflavone oxidation product (a phenoxyl radical) is not inert but can also promote oxidative damage itself [67]. Interestingly, the presence of ascorbate can reform the parent isoflavone from this intermediate allowing the isoflavone to act as an antioxidant in a “catalytic” manner which would also allow it to exert significant antioxidant effects *in vivo* at low concentrations [65, 67]. Finally, it is important to note that a key variable in assessing mechanisms of action is the fact that isoflavone preparations are not typically homogenous but contain complex mixtures of structurally distinct molecules. Moreover, it is now apparent that isoflavone metabolism can give rise to an array of products which themselves have different biological activities. For example, equol is produced by the action of gut microflora on ingested daidzein (see above). Interestingly, the composition of this microflora is not homogenous across the human population, and recent studies suggest that “equol producers” receive the health benefits of isoflavones consumption more than “equol nonproducers” [71]. In a similar fashion, reaction between isoflavones and reactive species *in vivo* can form novel isoflavone derivatives. For example, reaction with the RNS peroxynitrite or with the RCS hypochlorous acid form mono- or dinitrated or chlorinated isoflavones, respectively [61, 62]. We have shown that nitration and/or chlorination changes the antioxidant activity of the products compared to the parent isoflavones and in some cases increases antioxidant potency [62]. In this case, the first reactions would scavenge the reactive species but in addition also form more potent antioxidant isoflavones. We postulate that such mechanisms described above may reconcile the differences between dose-response relationships for antioxidant effects of isoflavones *in vivo* versus *in vitro* [72]. 

Attempts to produce animal diets free of phytoestrogens to provide more consistency in experiments designed to investigate the effects of added phytoestrogens had an unexpected, but critically important, effect. The animals showed had a marked increase in weight with the phytoestrogen-free diets, mostly in the form of abdominal fat [73]. This result suggested that phytoestrogens have a role in preventing the metabolic syndrome which in turn points to possible activity in PPAR signaling.

## 6. Isoflavones and Cell Signaling: Activation of PPARs

**Figure 4:**
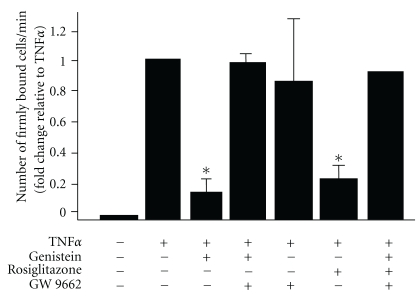
Isoflavones inhibit TNF*α*-induced monocyte adhesion to the vascular wall via activation of PPAR*γ*. Both genistein (1 *μ*M) and rosiglitazone (2 *μ*M) significantly reduced TNF*α*-induced monocyte adhesion to the vascular wall compared to TNF*α* alone. This inhibition was reversed by the PPAR*γ* antagonist, GW 9662 (5 *μ*M), revealing that genistein's inhibitory effect was PPAR*γ* dependent (reproduced with permission by the *American Journal of Physiology*).

Several studies have now developed the concept that activation of either PPAR*α* and/or PPAR*γ* is key to the biological effects of isoflavones [42]. This has been demonstrated in diverse experimental settings and cell types (including endothelium, monocytes, HepG2, bone marrow stromal cells) and importantly occurs at biologically relevant isoflavone concentrations. Using constructs containing either PPAR*α*-/PPAR*γ*- ligand binding-domains or sequences corresponding to promoter response elements, several independent studies [13, 42, 49, 74] have provided molecular evidence that isoflavones can stimulate PPAR*α*/*γ*- dependent gene expression. Importantly, this results in diverse functional effects that include modulating adipogenesis to regulating cellular responses to inflammation. Moreover, these cellular responses are inhibited by pharmacologically (using PPAR inhibitors) or molecularly (using siRNA-mediated downregulation of PPAR expression) based strategies to affect PPAR signaling [13, 14, 42, 49, 74]. The latter is critical, since the literature is replete with examples of putative PPAR ligands that subsequent studies have shown, in fact, to mediate cellular affects via PPAR-independent mechanisms. Figure 3 illustrates these points with data from our previous studies [13, 14] showing that in endothelial cells, isoflavones stimulate PPAR*γ*-dependent transcription of genes containing the PPAR*γ* response elements in their promoter, and this results in the inhibition of subsequent inflammatory cytokine (TNF*α*)-dependent monocyte rolling and adhesion (Figure 4). 

Interestingly, a survey of the literature does not reveal a clear association between the activation of either PPAR*α* or PPAR*γ* and the mediation of a biological response with evidence for both in mediating anti-inflammatory effects of isoflavones reported. For example red clover isoflavones inhibiting cytokine release from LPS activated macrophages via PPAR*α* [75]. Similarly, PPAR*α* activation has been discussed in the context of how isoflavones may prevent influenza [76]. On the other hand, anti-inflammatory effects have been shown to be PPAR*γ* dependent also including inhibition of amyloid-beta-dependent cytokine formation in astrocytes [77]. Our studies have shown that PPAR*γ*, but not PPAR*α*, is required for isoflavone-dependent inhibition of leukocyte rolling and adhesion to activated endothelial cells [13, 14] (Figure 4). Other reports in defined cell systems have also reported selective activation of one PPAR isoform and not the other. For example, methanolic (IF) extracts from soybean seeds stimulated transcriptional activity of PPAR*α*, but not PPAR*γ*, genes in monocyte U937 cells [78]. As the above discussion suggests, a detailed understanding of how isoflavones activate PPAR*α* or PPAR*γ* is lacking. It is clear, however, that isoflavones can activate both PPAR*α* and PPAR*γ*, and it is not surprising then that a number of studies have documented roles for these polyphenols in preventing diabetes and the metabolic syndrome with mechanisms ranging from improving lipid homeostasis to insulin sensitivity [49, 79–86]. 

**Figure 5:**
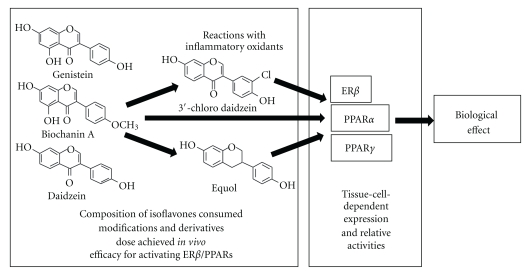
Regulation of isoflavone-dependent activation of PPAR signaling. Isoflavone consumption has been shown to activate ER*β*-, PPAR*α*-, or PPAR*γ*-dependent signaling pathways and in this way may exert control over diverse biological processes that are associated with health benefits of consuming these compounds. Summarized in this figure are the key factors that likely control how isoflavones modulate signaling. The core 7-hydroxy-benzopyran-4-one structure has recently been shown to be important for PPAR*α* and PPAR*γ* activation, and importantly this core is present in many plant-derived isoflavones that comprise natural and commercial preparations (shown are the structures of genistein, daidzein, and biochanin A as examples). Also indicated is the 7-hydroxy group on the A-ring that has been shown to be critical for PPAR*α* and PPAR*γ* activation [13, 14]). Modification of isoflavones, for example, via reactions with inflammatory oxidants can form a variety of halogenated isoflavones which in turn can alter the efficacy of isoflavone-dependent activation of signaling (shown as an example is 3′-chlorodaidzein). Note that multiple positional isomers of chlorine and bromine (other endogenous halogens and nitration products are possible [13, 61–63]. Moreover, gut microflora metabolism has been shown to be important in producing equol from daidzein and may have cardiovascular protective effects. We propose that a better understanding of isoflavone metabolism, the modifications that occur, and how they influence activation of ER/PPAR pathways is central to elucidating molecular mechanisms by which these compounds affect disease. Additional and important factors that will dictate the biological response to isoflavones include expression profiles and relative activities and interactions between of ER*β*-, PPAR*α*-, and PPAR*γ*-dependent pathways.

The discussion above serves to underscore the heterogeneity of responses elicited by isoflavone-mediated activation of PPARs. It remains unclear to date why in some cases both PPAR*α* and PPAR*γ* are activated, while in others why only one PPAR isoform is activated versus the other. Potential factors/variables that may modulate isoflavone-dependent activation of PPARs and signaling in general include the cell type, the presence/absence of PPAR co-activators, competition between ER*α* and PPAR signaling, and the dose and composition of isoflavones preparations (see Figure 5). For example, Dang elegantly showed that low concentrations (<1 *μ*M) of genistein stimulated osteogenesis whilst inhibiting adipogenesis in mesenchymal progenitor cells via ER mechanisms, whereas at slightly higher concentrations, the opposite response was observed which was mediated by PPAR*γ* activation [80]. Similarly, isoflavone-dependent activation of PPAR*γ* was shown to be important in the inhibition of estradiol-induced proliferation of uterine leiomyoma [57]. These latter examples highlight the potential for isoflavones to modulate estrogen signaling via indirect mechanisms and suggest a complex cross-talk between PPAR and ER signaling, that is regulated by isoflavones (Figure 5). With respect to isoflavones' type, studies have shown that several structurally distinct isoflavones can activate PPARs with similar efficacies [13]. It is not clear how the presence of different isoflavones would affect PPAR activation. If additive or synergistic, however, one can speculate that the effective dose of a given isoflavone to activate PPAR*α*/*γ* would be even lower in the context of a complex mixture as occurs during dietary exposure. In this scenario of exposure to multiple different isoflavones, we speculate that PPAR*α*/PPAR*γ* activation represents the primary signaling pathways affected by these compounds. Finally, we note that other factors may also modulate PPAR activation efficacy as illustrated by dietary exposure studies showing that soy protein alone increased PPAR*α*, but this response was increased further in the presence of isoflavones [87].

## 7. Remaining Questions and Future Perspectives

The potential role of PPARs to mediate biological actions of isoflavones is gaining appreciation. Less clear are the molecular mechanisms that are involved. Do isoflavones bind PPARs directly and/or do they affect PPAR signaling indirectly? Structure-activity relationship studies clearly suggest the former, but the latter possibility should also be considered. For example, oxidized fatty acids have been suggested to be potent PPAR*γ* agonists and isoflavones may influence these by affecting redox reactions. What controls the dual effects of isoflavones as PPAR*α* and PPAR*γ* agonists? What are the downstream targets of isoflavone-mediated PPAR activation, are they unique or do they overlap with PPAR activation by synthetic agonists? This is an intriguing question, since to our knowledge isoflavones are the only class of molecules that can activate both ER*β* and PPARs, raising the question of whether there is cross-talk between ER*β* and PPARs activation, and how this is regulated. Coupled with a better understanding of the potential for antagonistic, additive, or synergistic effects between structurally distinct isoflavones in activating PPARs, we feel that addressing these questions is likely to reveal novel insights into how these polyphenols influence diverse biological processes.
